# Global deletion of the immune cell transcription factor, T-bet, alters gut microbiota and insulin sensitivity in mice

**DOI:** 10.3389/fgene.2024.1502832

**Published:** 2024-11-27

**Authors:** E. Stolarczyk, C. T. Vong, N. Garrido-Mesa, E. Marks, D. Abdel-Aziz, Q. Ju, I. Jackson, N. Powell, G. M. Lord, J. K. Howard

**Affiliations:** ^1^ Diabetes and Obesity Theme, School of Cardiovascular and Metabolic Medicine and Sciences, King’s College London, London, United Kingdom; ^2^ School of Immunology and Microbial Sciences, King’s College London, London, United Kingdom

**Keywords:** T-bet, gut microbiota, insulin sensitivity, glucose homeostasis, fecal transfer, colonic immunity, short-chain fatty acids

## Abstract

The gut microbiota plays a role in energy homeostasis: its composition differs in lean and obese mice and may impact insulin sensitivity. The immune system has co-evolved with the gut microbiota, but direct regulation of microbial communities by the immune system and its metabolic impact is unclear. Mice lacking the immune cell specific transcription factor T-bet (Tbx21) are insulin sensitive. Compared with wild-type mice, *T-bet* deficient mice were found to have a higher proportion of colonic regulatory T cells despite significantly fewer colonic T cells, B cells and NK cells. Microbiota deletion by administration of antibiotics, increased colonic immune cell numbers. Furthermore, we report that *T-bet* ^*−/−*^ mice have an altered gut microbial composition and fecal short-chain fatty acid content, with an increase in butyrate production, compared with wild-type mice. Finally, in a proof-of concept study, we show that the enhanced insulin sensitivity observed in *T-bet* ^*−/−*^ mice is temporarily transmissible to antibiotic-treated wild-type mice through fecal transfer. Immune regulation of the gut microbiota by T-bet may be a novel pathway modulating insulin sensitivity.

## Introduction

Obesity has dramatically increased in recent decades and increases the risk of cardiovascular and other associated metabolic disorders (Chen et al., 2024; Powell-Wiley et al., 2021). Accumulating evidence from rodent models indicates that the gut microbiota plays a role in the regulation of fat storage, obesity associated inflammation and insulin resistance. The intestinal microbiota comprises over 10^13^ micro-organisms, including bacteria, archaea, eukaryotes and viruses, whose genomes collectively exceed the human genome in complexity by more than 300 to 400-fold (Li et al., 2014). The microbiota performs functions critical for host digestive efficiency, such as the digestion of complex plant carbohydrates and other dietary substances and influencing the development of the immune system (Maynard et al., 2012; Zhou et al., 2024). Recent evidence suggests that the microbiota may play a role in metabolic health and disease (Moreno-Indias et al., 2014; Bastos and Rangel, 2022). Indeed, obesity in mice appears to be associated with a gut microbiota that has capacity for energy harvest and which also appears transmissible (Turnbaugh et al., 2008; Backhed et al., 2004; Turnbaugh et al., 2006; Cheng et al., 2022). Modification of the microbiota with antibiotics and probiotics can improve the host energy homeostasis in rodents (Cani et al., 2007; Membrez et al., 2008), although the evidence in humans is less clear (Vallianou et al., 2020). While there was no impact on weight, it has been reported that transfer of gut microbiota from lean individuals was able to improve insulin sensitivity of individuals with metabolic syndrome (Vrieze et al., 2012).

Many factors, such as host diet and genetic background, determine microbiota composition (Parks et al., 2013; Scott et al., 2013; Šik Novak et al., 2023). The role of the microbial community in influencing the host immune system is well recognised (Hansen et al., 2012; Salvador et al., 2023). T-bet (Tbx21) is a member of the T-box transcription factor family, which regulates the differentiation and function of immune cells (Lazarevic et al., 2013). The expression of T-bet is limited to immune cells and is recognised to have a key role in many cells of the adaptive and innate immune system where it influences development, survival and effector functions (Lazarevic et al., 2013). Studies have shown that T-bet maintains mucosal homeostasis and impacts in cellular and molecular pathways in T cells, innate lymphoid cells (ILCs) and dendritic cells, which can impact the microbiota. Mice that lack T-bet on a background that lacks adaptive immunity, *Rag2* ^*−/−*^, develop ulcerative colitis in a microbiota dependent manner. Dysregulated tumour necrosis factor (TNF) production from colonic dendritic cells underlies this colitis by causing epithelial apoptosis. In dendritic cells Tbet was found to repress the TNF alpha gene, whereas in CD4^+^ T cells T-bet transactivates it (Garrett et al., 2007). Importantly, this disease was found to be transmissible to wild-type (WT) mice by transfer of the *T-bet* ^*−/−*^ x *Rag2* ^*−/−*^ microbiota when a pathobiont is present (Garrett et al., 2007) suggesting that the immune system may play a role in regulating the gut microbiota. Indeed, *Helicobacter typhlonius* was later identified as responsible in triggering the inflammation in the *T-bet* ^*−/−*^ x *Rag2* ^*−/−*^ mice, an effect that was dependent on interleukin-7 receptor positive innate lymphoid cells (Powell et al., 2012).

We have previously described a favourable metabolic phenotype of the T-bet deficient mice, supporting a role for T-bet as a metabolic regulator. *T-bet* ^*−/−*^ mice display increased visceral adiposity compared with WT mice and yet are more insulin sensitive (Stolarczyk et al., 2013). The transcription factor T-bet is known to drive the expression of CXCR3 on T cells and deletion of T-bet expression leads to a reduction of immune cell infiltration in the adipose tissue (Lord et al., 2005). Indeed, global deletion of T-bet changes the immune cell subpopulations within visceral adipose tissue and impacts adipose tissue inflammation. We have also shown that the metabolic phenotype of the *T-bet* ^*−/−*^ mice mapped to the adaptive immune system (Stolarczyk et al., 2013). Work on colonic homeostasis has highlighted the role of the adaptive immune system in influencing microbiota composition (Zhang et al., 2015). However, the role of T-bet expression in the regulation of the gut microbiota and its impact on adiposity and glucose homeostasis is unknown.

## Materials and methods

### Animals

Male BALB/c wild-type (WT), *T-bet* ^*−/−*^ mice, were bred in the King’s College London Biological Service Unit, and housed in a specific pathogen-free environment. Studies were carried out according to the UK Home Office guidelines. The mice had free access to food and water, with a High Fat Diet (HFD, 58% calories from fat, ref D12331) feeding regime started at 8 weeks of age for 20 weeks (Research Diet Inc., United States). In order to deplete gut microbiota, mice were treated as described previously (Garrett et al., 2007). Alterations are as followed: ampicillin (0.5 g/L; Roche), vancomycin (0.5 gm/l; Sigma), neomycin sulfate (0.5 g/L; Sigma), and metronidazole (0.5 g/L; Sigma) were dissolved in 0.45 µm filtered drinking water and fluid intake was monitored. Treatment was started 2 weeks prior to HFD feeding and continued over the 20 weeks of diet. Body weights and immune cell populations in adipose tissue on male BALB/c wild-type (WT) and *T-bet* ^*−/−*^ mice fed HFD not treated with antibiotics have previously published in (Stolarczyk et al., 2013) and some of the results of the HFD antibiotic treated mice are compared with these historic non antibiotic-treated controls.

Due to breeding constraints, age and sex-matched mice were studied in rolling groups of no fewer than 3-4 per genotype per group at the same time of day and, as there was no significant difference in data obtained, results were pooled. Non-fasted mice were sacrificed by CO_2_ inhalation and blood was collected by terminal cardiac puncture. Plasma was stored at −20°C until analysed. Tissues were snap frozen for further analysis or kept at 4°C prior to cell extraction and organ culture.

### Microbiota transfer

Six-week-old BALB/c WT males were given antibiotics for 2 weeks in drinking water with modification of previously described protocol (Garrett et al., 2007). Mice were treated with a mix of ampicillin (0.5 g/L; Roche), vancomycin (0.5 g/L; Sigma), neomycin sulfate (0.5 g/L; Sigma) and metronidazole (0.5 g/L; Sigma) dissolved in 0.45 µm filtered drinking water and fluid intake was monitored. The fecal content from an untreated donor mouse was homogenised in 3 mL of PBS and was homogenised in 3 mL of PBS and filtered on 70 μm filter; 0.2 mL of the solution was given by oral gavage per mouse. Following the gavage, the mice were housed in top filtered cages to avoid cross-contamination. The new microbiota was left to establish for 2 weeks before any *in vivo* studies were performed.

### *In vivo* studies

Mice were fasted overnight, and tail vein blood was collected. Plasma samples were stored at −20°C until analysed. Intraperitoneal glucose tolerance tests (IPGTT, 1.5 g glucose/kg body weight, Sigma, United Kingdom) and insulin tolerance tests (ITT, 1 U insulin/kg body weight, Actrapid, United Kingdom) were performed as previously described (Howard et al., 2004).

### Analysis of metabolic parameters

Blood glucose was measured using a glucometer (Statstrip Xpress, Nova Biomedical, United Kingdom). Plasma insulin and leptin concentrations were determined by enzyme-linked immunosorbent assay (ELISA) (Crystal Chem, United States) as previously described (Howard et al., 2004). Homeostasis model assessment of insulin resistance (HOMA-IR) was calculated from the product of fasting serum glucose (mmol/L) and insulin (mU/mL), and then divided by 22.5 (Matthews et al., 1985).

### Isolation of mononuclear cells and flow cytometry

The stromal vascular fraction (SVF) containing mononuclear cells and pre-adipocytes was extracted from adipose tissue as previously described (Stolarczyk et al., 2013) and colonic immune cells were isolated following enzymatic digestion as previously described (Powell et al., 2012). Cells were stained with antibodies conjugated to fluorochromes, CD45 (30-F1), CD3 (145-2C11), CD4 (GK1.5), CD8 (53–6.7), NKp46 (29A1.4), B220 (RA3-6B2), CD11b (M1/70), F4/80 (BM8), Foxp3 (FJK-16s) (eBiosciences, United Kingdom). 7-Amino-actinomycin D (eBiosciences, United Kingdom) or LIVE/DEAD Fixable Dead Cell Stain (Invitrogen, United Kingdom) were utilised to discriminate between live and dead cells. Samples were acquired using a LSRII cytometer (Becton Dickinson, United States) and data was analysed using FlowJo software (Tree Star, United States).

### Fluorescent *in situ* hybridisation (FISH) combined with flow cytometry (FISH-flow)

Fluorescent *in situ* hybridisation (FISH) combined with flow cytometry was performed as previously described (Alhabbab et al., 2015).

### Microbial metabolite analysis

Fecal samples were weighed and suspended in 500 μL of water with 0.5% phosphoric acid and frozen at −20°C immediately after collection. Once thawed, the fecal suspensions were homogenized, 0.05 mg of 4-Me-Valeric acid was added as internal standard and the organic layer was extracted by centrifugation after addition of 1 mL of ethyl acetate. The GC-MS system consisted of an Agilent HP6890 Series GC with HP 7683 Series injector (Agilent Technologies, Palo Alto, CA, United States) coupled to an Agilent HP 5973 mass selective detector. The GC was fitted with a high polarity, polyethylene glycol (PEG), fused silica capillary column DB-WAXETR (30 m, 0.25 mmid, 0.25 μm film thickness). Injection was made with a volume of 3 μL and an injector temperature of 250°C. Identification of the SCFAs was based on the retention time of standard compounds and the analysis was performed on the Agilent MSD ChemStation D.03.00.611.

### Statistical analyses

Results are expressed as mean ± S.E.M. Non-parametric data were analysed using a Mann-Whitney U test or two-way analysis of variance (ANOVA), as appropriate, using GraphPad Prism (GraphPad Inc., United States).

## Results

**FIGURE 1:**
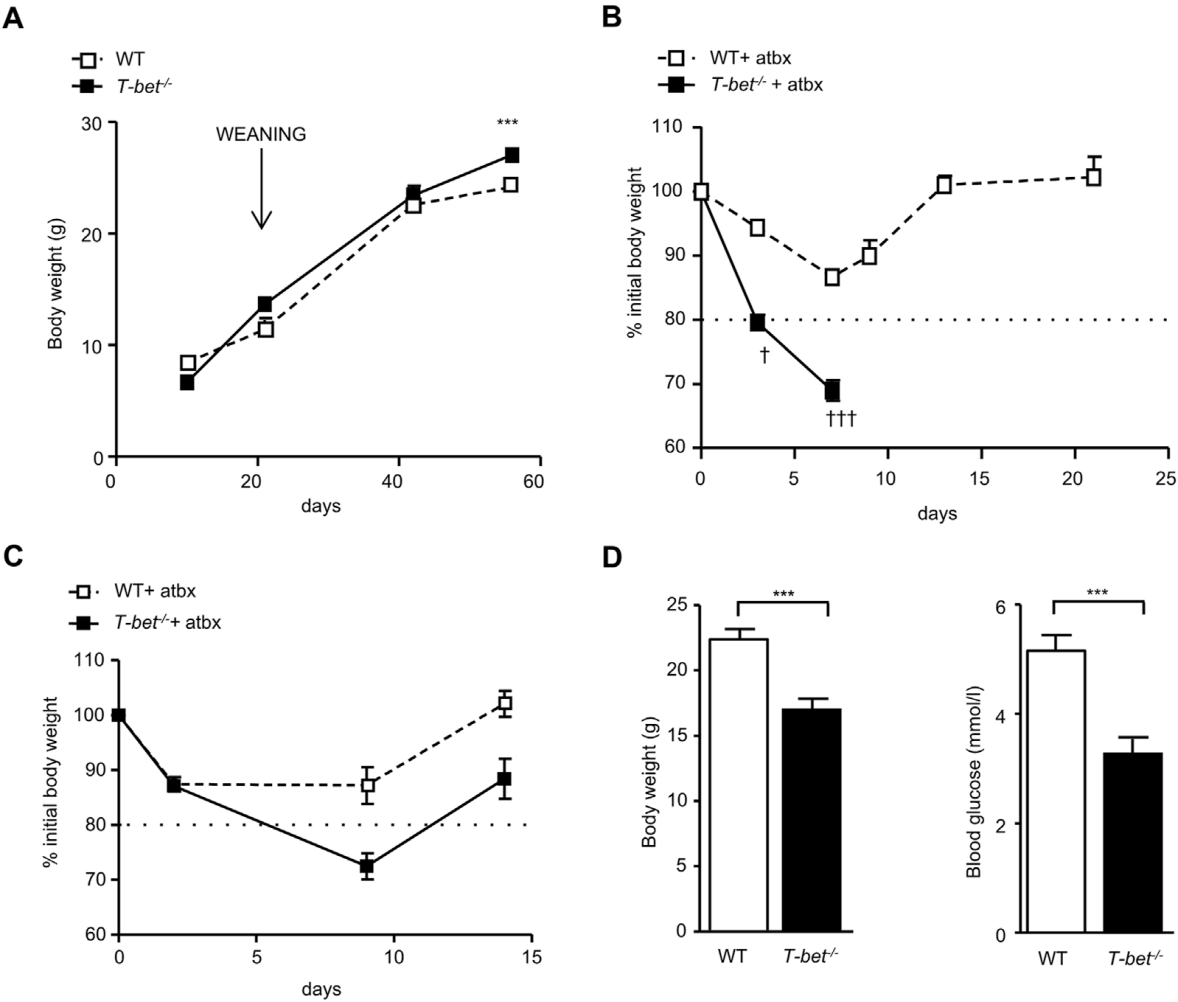
Greater weight loss and lower glucose levels in antibiotic treated chow fed *T-bet* ^*−/−*^ mice **(A)** Body weight of WT and *T-bet* ^*−/−*^ mice from 10 days to 8-weeks-old (n = 4–10). **(B)** Body weight change of 10–13-week-old WT and *T-bet* ^*−/−*^ mice during treatment with broad spectrum antibiotics (n = 8–15). † indicates mice that died or needed to be culled due to profound weight loss. **(C)** Body weight change of 6-week-old WT and *T-bet* ^*−/−*^ mice during treatment with antibiotics (n = 10). **(D)** Body weight and non-fasting glucose levels following antibiotic treatment of 6-week-old WT and *T-bet* ^*−/−*^ mice (n = 8). Data represent means ± SEM. ***p < 0.005.

**FIGURE 2:**
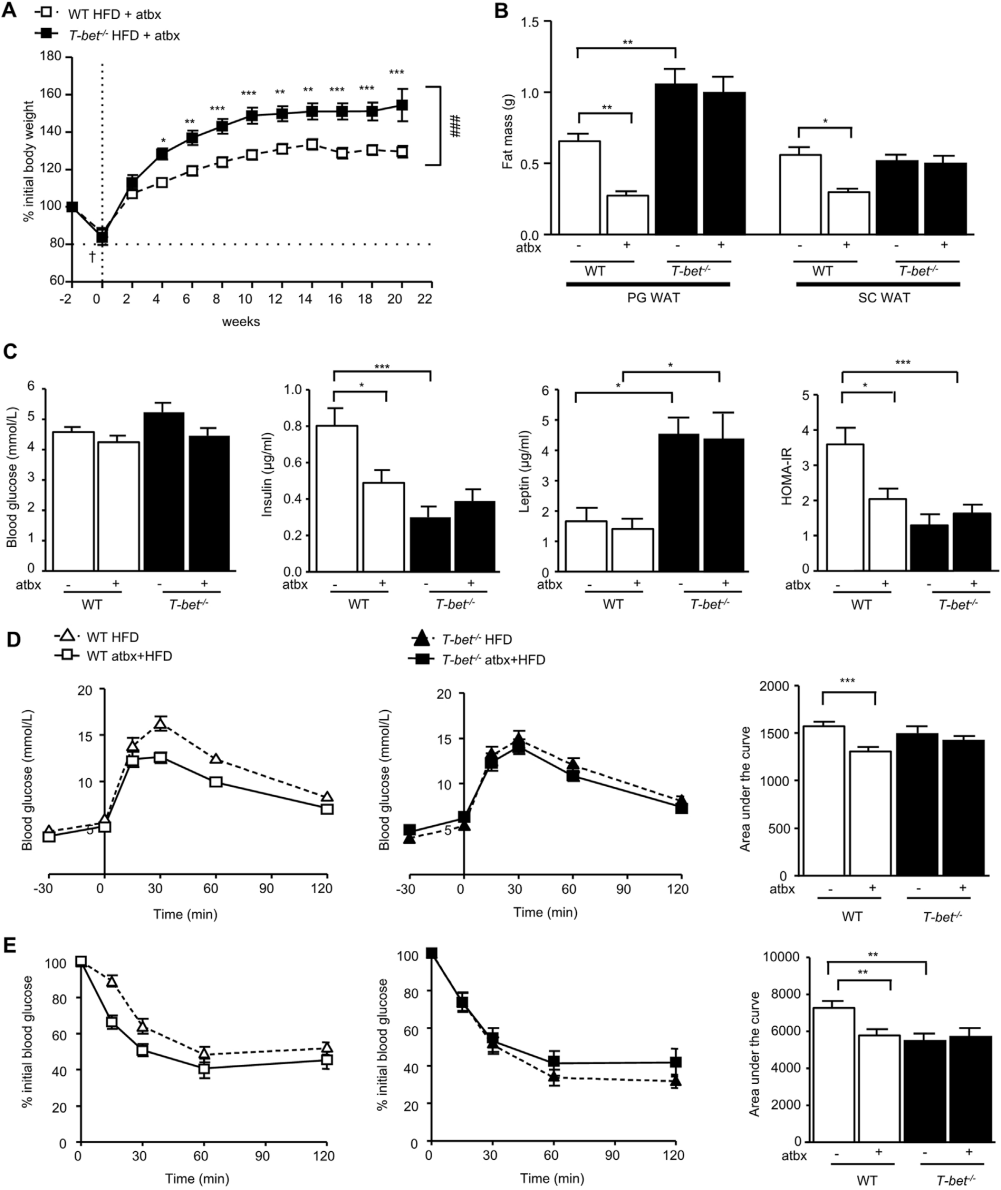
Antibiotic-induced depletion of microbiota reduces fat mass and improves insulin sensitivity of HFD-fed WT but not *T-bet* ^*−/−*^ mice. **(A)** Body weight of WT and *T-bet* ^*−/−*^ mice during 20 weeks of HFD with antibiotics (atbx) (n = 15–17). **(B)** Weights of perigonadal adipose tissue (PG WAT) and subcutaneous adipose tissue (SC WAT) fat pads from WT and *T-bet* ^*−/−*^ mice after 20 weeks of HFD with and without antibiotics (n = 15–17) **(C)** Fasting glucose, insulin and leptin levels and HOMA-IR in WT and *T-bet* ^*−/−*^ mice after 14 weeks of HFD with and without antibiotics (n = 12–15). **(D)** Glucose tolerance test of WT and *T-bet* ^*−/−*^ mice after 9 weeks of HFD with and without antibiotics and the corresponding area under the curve (n = 13–16). **(E)** Insulin tolerance test of WT and *T-bet* ^*−/−*^ mice after 10 weeks of HFD with and without antibiotics and the corresponding area under the curve (n = 13–16). Data represent means ± SEM. ∗*p* < 0.05, ∗∗*p* < 0.01, ***p < 0.005, Two-way ANOVA.

The gut, typically sterile until birth, is gradually colonised by micro-organisms over the following weeks (Wall et al., 2009). At 8 weeks old *T-bet* ^−/−^ mice were significantly heavier than WT mice (Stolarczyk et al., 2013) but, prior to weaning at the age of 3 weeks, the body weights of the genotypes were similar (Figure 1A). The divergence in body weight between the genotypes therefore appeared to parallel the timeline for establishment of the gut microbiota. In order to investigate a potential role of the gut microbiota in the metabolic phenotype of the T-bet deficient mouse, we administered broad-spectrum antibiotics (ampicillin neomycin, metronidazole and vancomycin) to *T-bet* ^*−/−*^ and wild-type mice to deplete their microbiota. The initial stages of antibiotic treatment in mice can result in weight loss, before recovery (Reikvam et al., 2011). Indeed, the weight loss was mild and temporary in the 10-week-old antibiotic-treated WT cohort (Figure 1B). In contrast, administration of antibiotics caused such profound weight loss in the *T-bet* ^*−/−*^ cohort that they either died or had to be culled due to >20% body weight loss, as per animal welfare regulations (Figure 1B). Post-mortem examination of the *T-bet* ^*−/−*^ mice failed to reveal any overt pathology, other than a profound loss of body fat. As *T-bet* ^*−/−*^ mice are already known to be more insulin sensitive than WT mice (Stolarczyk et al., 2013), we wondered whether the antibiotic-induced weight loss might have resulted in lethal hypoglycaemia in this genotype. Antibiotic depletion of microbiota in a further group of 6-week -old mice was also accompanied by weight loss did indeed reveal significantly lower blood glucose levels in *T-bet* ^*−/−*^ compared with WT mice (Figures 1C,D). Increased survival of antibiotic treated *T-bet* ^*−/−*^ mice was achieved by increasing energy intake. Feeding all the antibiotic-treated mice with a high fat diet (HFD), allowed the analysis and comparison of the long-term effect of microbiota depletion on body weight and glucose homeostasis in both genotypes, WT and *T-bet* ^*−/−*^ mice, over 20 weeks (Figure 2). Depletion of microbiota was confirmed by enumeration of bacteria after 20 weeks by fluorescent *in situ* hybridisation coupled with flow cytometry (FISH-Flow) together with an observed increase in cecal size, a common feature of a germ-free-like phenotype in mice (Reikvam et al., 2011) (Supplementary Figure S1A, B).

HFD-fed antibiotic treated *T-bet* ^*−/−*^ mice gained significantly more weight than the wild-type controls (Figure 2A). We have previously reported that HFD-fed *T-bet* ^*−/−*^ mice on a BALB/c background without antibiotic treatment have increased visceral adiposity compared with HFD-fed WT mice (Stolarczyk et al., 2013). We therefore evaluated the weight of the fat pad depots between the antibiotic-treated HFD-fed WT mice and *T-bet* ^*−/−*^ mice and compared the results to those of age and sex-matched mice of both genotypes on the HFD that had not received antibiotic treatment, as previously reported (Stolarczyk et al., 2013). Whereas antibiotic treatment of HFD-fed WT mice was associated with a significant reduction in fat mass, antibiotic-induced depletion of microbiota did not affect adiposity in HFD-fed *T-bet* ^*−/−*^ mice (Figure 2B). Adipocytes of HFD *T-bet* ^*−/−*^ mice were significantly larger than those of WT mice (Stolarczyk et al., 2013). This difference in adipocyte size persisted when the mice were treated with antibiotics (Supplementary Figure S2A, B) and was associated with higher circulating leptin levels than that of WT mice (Figure 2C).

We next examined the effect of antibiotic-induced microbiota depletion on glucose homeostasis. Fasting glucose levels were not significantly different between the genotypes with or without antibiotic treatment (Figure 2C). However, in addition to the antibiotic-induced reduction in fat mass in WT mice, antibiotic treatment also significantly lowered the fasting insulin level in HFD WT mice but did not further reduce the lower level observed in *T-bet* ^*−/−*^ mice (Figure 2C). Glucose tolerance and insulin sensitivity of WT mice was also improved following 20 weeks of antibiotic treatment (Figures 2D,E). However, the already enhanced glucose homeostasis observed in HFD-fed T-bet deficient mice (Stolarczyk et al., 2013) was not further improved by antibiotic-induced deletion of the microbiota (Figures 2D,E). Overall, WT mice treated with antibiotics had improved glucose homeostasis, but whereas this improvement in insulin sensitivity in WT mice was accompanied by a reduction in fat mass, the fat mass in the already insulin sensitive *T-bet* ^*−/−*^ mice remained significantly greater.

**FIGURE 3:**
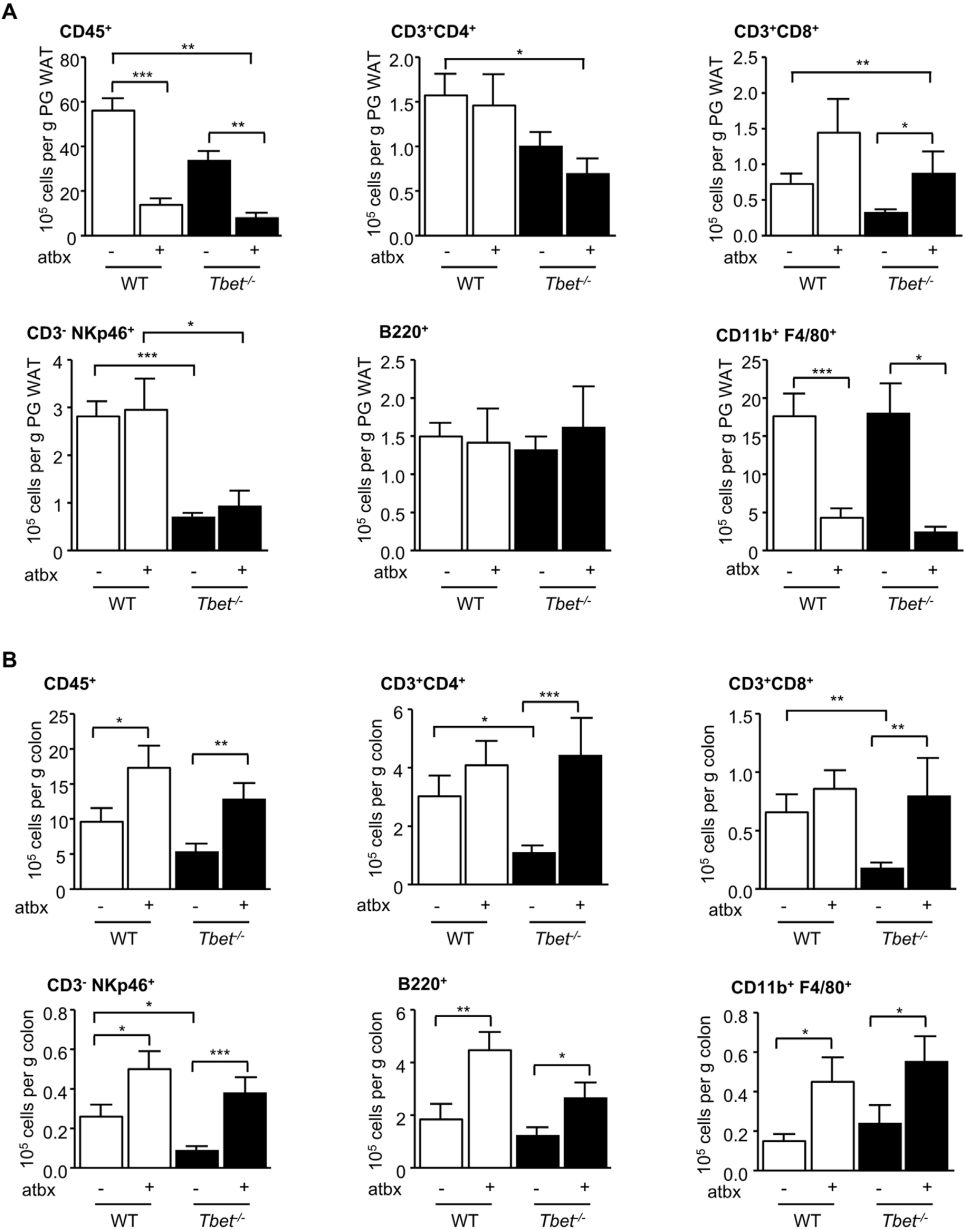
Microbiota depletion modified adipose tissue and colonic immunity. Flow cytometric analyses of immune cells extracted from **(A)** PG WAT and **(B)** colon of WT and *T-bet* ^−/−^ mice after 20 weeks of HFD with and without antibiotics (atbx). Number of immune cells (CD45^+^), CD3^+^ CD4^+^ T cells, CD3^+^ CD8^+^ T cells, B cells (CD19^+^), NK cells (CD3^−^ NKp46^+^), and macrophages (CD11b^+^ F4/80^+^) are expressed per Gram of PG WAT and colon (n = 8–22). Data represent means ± SEM. *p < 0.05, **p < 0.01, ***p < 0.005.

We have previously reported fewer total (CD45^+^) immune cells in the peri-gonadal adipose tissue of *T-bet* ^*−/−*^ mice compared with WT mice (Stolarczyk et al., 2013). Gut microbiota depletion further decreased the total immune cell number in the visceral adipose tissue of both genotypes (Figure 3A). The CD11b^+^ F4/80^+^ macrophages were specifically reduced by antibiotic treatment in both *T-bet* ^*−/−*^ and WT mice, with little impact of this treatment on the numbers of CD3^+^CD4^+^, CD3^−^NKp46^+^ and B220^+^ cells. CD3^+^CD8^+^ cells in the peri-gonadal adipose tissue depot were increased in number with antibiotic treatment in both genotypes (Figure 3A). Gut microbiota depletion is known to increase the number of immune cells in the colon (Wlodarska et al., 2011). The microbiota is also involved in the maturation and the stability of the colonic immunity (Hooper et al., 2012). As with peri-gonadal white adipose tissue, the number of T cells (CD3^+^CD4^+^ and CD3^+^CD8^+^), B cells (B220^+^) and NK cells (CD3^−^NKp46^+^) were significantly lower in the colons from untreated *T-bet* ^*−/−*^ mice compared with WT mice (Figure 3B). Antibiotic treatment caused an increase in the number of total immune cells and all subpopulations examined in both genotypes. The number of these immune cell subpopulations in the colon was similar following microbiota depletion and was independent of genotype (Figure 3B), reflecting the importance of the gut microbiota to the immune status of the colon.

**FIGURE 4:**
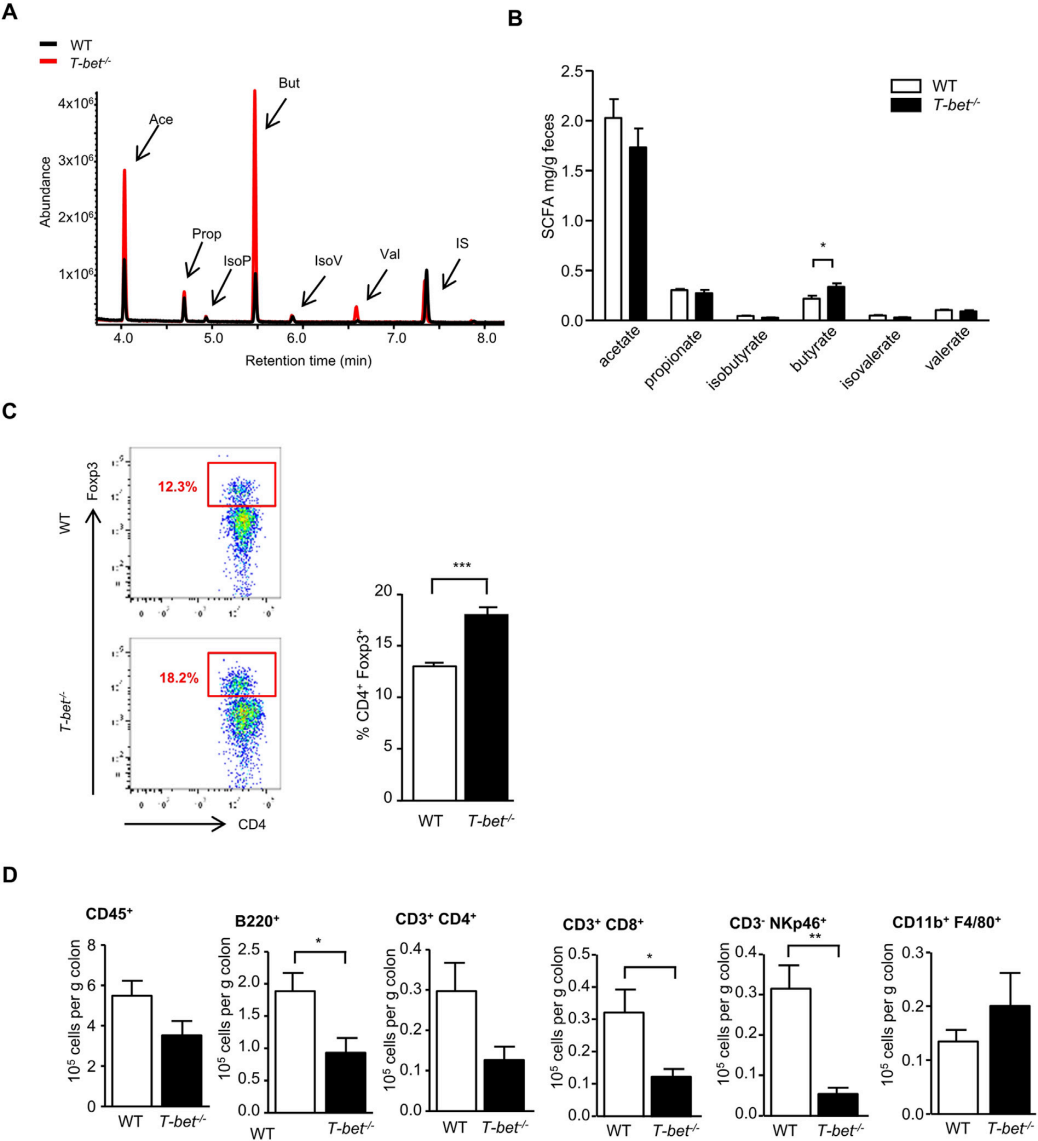
T-bet deficiency alters gut microbiota and fecal short chain fatty acid composition and impacts colonic immunity **(A)** Representative GC-MS spectrum of organic acids analysis (SCFA) from 8-week-old WT and *T-bet* ^*−/−*^ mice. **(B)** Short-chain fatty acid composition of feces from 8-week-old chow fed WT and *T-bet* ^*−/−*^ mice (n = 8). **(C)** Representative FACS plot identifying Foxp3^+^ cells within CD4^+^ population in cells extracted from the colon and the corresponding quantification in WT and *T-bet* ^*−/−*^ mice (n = 7). **(D)** Flow cytometric analyses of immune cells extracted from colon of 8-week-old chow fed WT and *T-bet* ^−/−^ mice. Number of immune cells (CD45^+^), CD3^+^ CD4^+^ T cells, CD3^+^ CD8^+^ T cells, B cells (B220^+^), NK cells (CD3^−^ NKp46^+^), and macrophages (CD11b^+^ F4/80^+^) are expressed per Gram of colon (n = 7). Data represent means ± SEM. *p < 0.05, **p < 0.01, ***p < 0.005.

Short-chain fatty acids (SCFA), produced by gut microbial fermentation of soluble fibre in the distal gut, are an important energy source for the host (den Besten et al., 2013) and may play a role in regulation of energy homeostasis and insulin sensitivity through activation of G protein coupled receptors (GPCRs) receptors, GPR43 and GPR41, also known as free fatty acid receptors FFAR2, FFAR3, respectively (Puddu et al., 2014). The SCFA butyrate, in particular, has been linked with improved insulin sensitivity (Gao et al., 2009). We therefore hypothesised that T-bet deficiency in mice would alter the colonic microbiota and SCFA in a manner which may enhance insulin sensitivity. Consistent with reports linking these commensals with SCFA production and colonic immunity (Smith et al., 2013), we observed an increase in butyrate production in *T-bet* ^*−/−*^ compared to WT mice (Figures 4A, B). Butyrate production has been linked to the development of peripheral anti-inflammatory T-regulatory cells (Tregs) and in the regulation of colonic Tregs homeostasis (Smith et al., 2013; Atarashi et al., 2013; He et al., 2024). Previously, we have described an increase in the percentage of infiltrating CD4^+^Foxp3^+^ cells in the visceral adipose tissue of T-bet deficient mice (Stolarczyk et al., 2013) and we observed the same augmentation in the proportion of colonic CD4^+^Foxp3^+^ Tregs in *T-bet* ^*−/−*^ compared to WT mice (Figure 4C). As in the visceral adipose tissue (Stolarczyk et al., 2013), young *T-bet* ^*−/−*^ mice also had reduced immune cells numbers in the colon (CD3^+^ CD4^+^, CD3^+^ CD8^+^ CD3^−^NKp46^+^ and B220^+^) compared with wild-type mice (Figure 4D). Collectively, these results support a potential correlation between T-bet, the microbiota, inflammation and insulin sensitivity.

**FIGURE 5:**
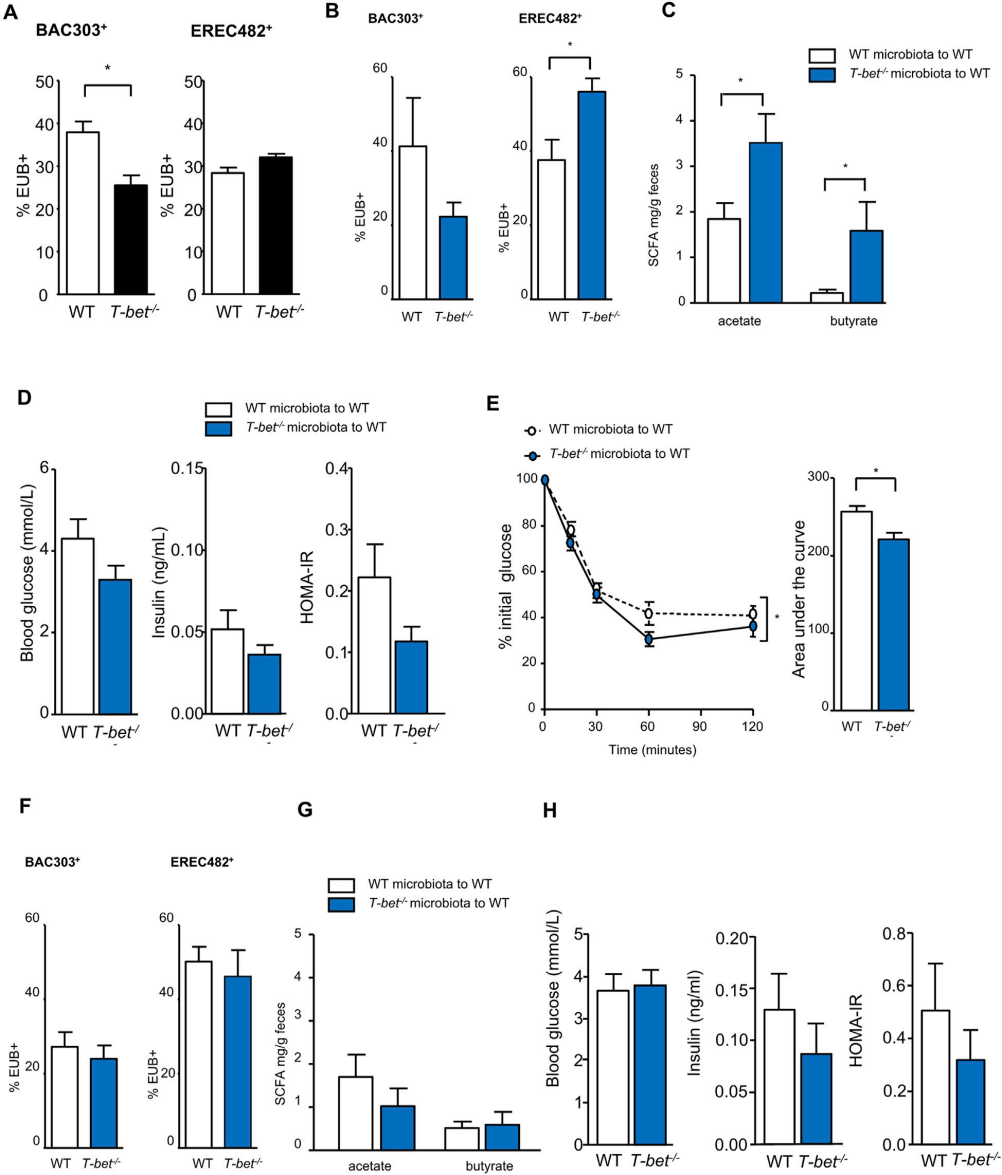
Transfer of T-bet deficient microbiota improves insulin sensitivity in wild-type mice **(A)** Microbiota composition characterisation by FISH-flow analysis for the Bac303 and Erec482 targeted groups in 8-week-old chow fed WT and *T-bet* ^*−/−*^ microbiota. Shown in black to indicate baseline microbiota of the two genotypes. (n = 5). (B to H data shown in blue to indicate fecal transfer to WT host from the two genotypes). **(B)** Microbiota composition characterisation by FISH-flow for the Bac303 and Erec482 targeted groups 2 weeks following the transfer of WT and *T-bet* ^*−/−*^ microbiota to WT host (n = 5). **(C)** Short-chain fatty acid composition (acetate and butyrate) in feces 2 weeks following the transfer of WT and *T-bet* ^*−/−*^ microbiota to WT host (n = 5). **(D)** Fasting glucose and insulin levels, and HOMA-IR from WT mice 2 weeks following the transfer of WT and *T-bet* ^*−/−*^ microbiota to WT host (n = 5). **(E)** Insulin tolerance test and corresponding area under the curve from WT hosts following the transfer of WT and *T-bet* ^*−/−*^ microbiota to WT host (n = 5). **(F)** Microbiota composition characterisation by FISH-flow for the Bac303 and Erec482 groups 5 weeks following the transfer of WT and *T-bet* ^*−/−*^ microbiota to WT host (n = 5). **(G)** Short-chain fatty acid composition (acetate and butyrate) in feces 4 weeks following the transfer of WT and *T-bet* ^*−/−*^ microbiota (n = 5). **(H)** Fasting glucose and insulin levels and HOMA-IR from WT mice 5 weeks following the transfer with WT and *T-bet* ^*−/−*^ microbiota to WT host (n = 5). Data represent means ± SEM. ∗*p* < 0.05.

Microbiota composition is influenced by mucosal immunity. Using FISH-Flow analysis, we first established that young *T-bet* ^*−/−*^ mice have altered colonic microbiota composition compared with WT mice. *T-bet* ^*−/−*^ mice were found to have proportionally fewer *Bacteroides* and *Prevotella* (as measured using the Bac303 FISH probe) and more Lachnospiraceae (Erec482 probe) bacteria compared with WT mice (Figure 5A), reflecting the Bacteroidetes and Firmicutes phyla, respectively (Bervoets et al., 2013). In order to investigate the role of microbiota on the metabolic phenotype observed in *T-bet* ^*−/−*^ mice, we decided to cross-transfer colonic microbiota from WT and *T-bet* ^*−/−*^ mice by fecal gavage to a WT host whose intestinal microbial communities had been depleted by 2 weeks administration of broad-spectrum antibiotics. FISH-Flow was then used dynamically to track the microbiota over 5 weeks and determine whether these differences in *T-bet* ^*−/−*^ colonic microbiota were transmissible to WT mice. In parallel with this we evaluated any associated changes in SCFA and insulin sensitivity following the microbiota transfer.

Two weeks following transfer of *T-bet* ^*−/−*^ microbiota to WT mice a similar colonic microbiota profile to that found in *T-bet* ^*−/−*^ mice was observed in the WT hosts (Figure 5B) with a significant increase in the Lachnospiraceae bacteria as detected by the Erec482 probe. This was associated with a significant increase in the acetate and butyrate content of the feces (Figure 5C). There was also a tendency for *T-bet* ^*−/−*^ microbiota to lower plasma glucose and insulin levels (Figure 5D) and insulin sensitivity of recipient WT mice was modestly but significantly enhanced 3 weeks post *T-bet* ^*−/−*^ microbiota transfer (Figure 5E). However, these effects on the microbiota, SCFA and glucose homeostasis were not sustained 5 weeks following transfer (Figures 5F–H).

Although the mice transferred with *T-bet* ^*−/−*^ derived microbiota tended to gain slightly more weight compared with those transferred with WT microbiota, this was not significant (Figure 5A). Moreover, transfer of a T-bet insulin sensitive microbiota did not have any effect on adipose tissue mass of a WT host (Figure 5H).

## Discussion

There is increasing recognition the gut microbiota may play a role in the development of inflammation, obesity and associated metabolic disease, particularly in rodents (Zhou et al., 2024; Lozupone et al., 2012). Previously, we have demonstrated that the mice on a BALB/c background, deficient for the transcription factor T-bet, were more insulin sensitive despite an increase in their visceral adipose tissue mass (Stolarczyk et al., 2013). This was accompanied by a reduction of the inflammation of the adipose tissue and an increase in the number and proportion of adipose regulatory T cells (Tregs). Our results indicate that the immune cell transcription factor T-bet may impact insulin sensitivity through regulation of the gut microbiota.

At weaning, *T-bet* ^*−/−*^ mice have the same body weight as the WT mice, and they only start to diverge when their gut microbiota is fully established at 8 weeks old. It has been suggested that early gut colonisation shapes future immune responses of the host, and colonization of germ free (GF) mice at 3 weeks of age with microbiota results in pro-inflammatory tuning of the immune system, which is not seen at 1 week old (Hansen et al., 2012). When antibiotic-induced microbiota depletion was performed in WT mice all of the mice recovered their initial body weight within 2 weeks, irrespective of the age of the mice at the start of the study. However, the outcome of the microbiota depletion was influenced by the age of the *T-bet* ^*−/−*^ mice at the initiation of antibiotic treatment. Most of the *T-bet* ^*−/−*^ mice receiving antibiotics at the age of 10 weeks old showed a profound weight loss and had to be culled (as per Home Office regulations for animal welfare with greater than 20% weight loss) or died within a week. However, when antibiotic treatment was started earlier, at 6 weeks old, the microbiota depletion induced only temporary, and less profound body weight loss and hypoglycaemia in *T-bet* ^*−/−*^ mice. Microbial colonisation of the gut occurs over this time frame and the development of colonic immunity and immune system maturation is known to occur in parallel. Whether this period of establishment of the microbial communities in the T-bet mice in particular accounts for the profound weight loss observed in this genotype treated with antibiotics is unclear. Indeed, microbiota manipulation has been shown to affect glucose homeostasis without affecting long-term weight development and colonic immunity (Bech-Nielsen et al., 2011).

As the impact of manipulation of the microbiota of *T-bet* ^*−/−*^ mice by antibiotics appeared to be age dependent, in terms of weight loss, compared to WT mice, we decided to compare the microbiota composition of chow-fed *T-bet* ^*−/−*^ and WT mice at 8 weeks old when the gut microbial composition and colonic immune system was likely more established. Using the Bac303 FISH probe and the Erec482 probe, young *T-bet* ^*−/−*^ mice were found to have proportionally less *Bacteroides* and *Prevotella* (the Bacteroidetes phylum) and more Lachnospiraceae (Firmicutes phylum) bacteria compared with WT mice. Obese mice have been described as having less Bacteroidetes compared with lean mice (Turnbaugh et al., 2006; Ley et al., 2005; Koliada et al., 2017; Trikha et al., 2021). However, the obese mice analysed in these studies were also insulin resistant compared to their lean counterparts, which is not the apparent phenotype of *T-bet* ^*−/−*^ mice. Importantly, such phylogenetic comparisons do not take in to account the broad differences that exist between members of the same phyla at the species level. We have previously highlighted this unusual phenotype, the uncoupling obesity and insulin resistance in *T-bet* ^*−/−*^ mice in a previous study, (Stolarczyk et al., 2013).

Interestingly, we have now found that the production of SCFAs is modified in *T-bet* ^*−/−*^ mice. The colonic microbiota from *T-bet* ^*−/−*^ mice were found to be producing more butyrate. This is consistent with the greater proportion of Lachnospiraceae detected with FISH-Flow in this study as these are likely to include butyrate producers. This increase in butyrate production may be also responsible for the observed increase in colonic Tregs, as has been described by others (Smith et al., 2013). However, as we have reported in visceral adipose tissue (Stolarczyk et al., 2013), *T-bet* ^*−/−*^ mice overall have fewer immune cells in their colon compared with that observed in WT mice. These differences in colonic immunity may impact and account for our observed differences in microbiota between the *T-bet* ^*−/−*^ and WT mice, as host adaptive immunity has been reported to alter microbiota composition (Zhang et al., 2015; Wang et al., 2023).

It has been suggested that homeostasis of Tregs can be driven by the composition of colonic microbiota and SCFA production, and effect that was dependant on FFAR2 expression on Tregs (Smith et al., 2013; Ostadmohammadi et al., 2022). However, these results are not supported by another report that highlighted the independence of FFAR2 and FFAR3 expression in IL-10 production by CD4^+^ T cells (Park et al., 2015). Indeed, FFAR2 and FFAR3 have not only been described on Tregs, but also on epithelial cells where they are able to modulate intestinal inflammation (Kim et al., 2013; Lednovich et al., 2022). They are also expressed on enteroendocrine cells, where they participate in the energetic metabolism by regulation of GLP-1 secretion, and subsequently regulation of glucose homeostasis (Tolhurst et al., 2012).

As microbiota can affect adipose tissue inflammation (Geurts et al., 2014; Wu et al., 2022; May and den Hartigh, 2021), the favourable phenotype observed in *T-bet* ^*−/−*^ mice may be at least partially linked with their microbiota composition and functional activities. Indeed, by depleting the microbiota in HFD fed mice, we found that in WT mice antibiotic treatment resulted in a reduction of adipose tissue mass which was accompanied by improved insulin sensitivity. Interestingly antibiotic treatment of *T-bet* ^*−/−*^ mice did not impact the fat mass of this genotype. However, while antibiotic depletion of the microbiota enhanced the insulin sensitivity of WT mice, it did not further increase the insulin sensitivity of the *T-bet* ^*−/−*^ mice in the HFD study. The reason for this genotypic difference is unclear. It may be that the microbiota populations present in the WT mice (which are not present in the already sensitive *T-bet* ^*−/−*^ mice) contribute more to the insulin resistance displayed by this HFD fed model and so the loss of these populations with antibiotic treatment improve the insulin sensitivity of this genotype. It likely that the mechanism of enhanced insulin sensitivity of *T-bet* ^*−/−*^ mice is not merely restricted to the differences in their gut microbiota. The global deletion of T-bet is known to affect immune cell subpopulations across a number of insulin sensitive tissues, including adipose tissue, as we have previously reported (Stolarczyk et al., 2013). Although these differences in adipose tissue immune cell subpopulations may be secondary to microbiota differences between the genotypes (particularly as these differences were observed within the visceral adipose tissue depot rather than the subcutaneous depot) the impact of T-bet deficiency on adipose tissue immune cells and inflammation may be independent of the microbiota. In this study we therefore compared the impact of antibiotic depletion of the gut microbiota on the immune cell composition of perigonadal adipose tissue (a type of visceral fat) and that of the colon. The antibiotic treatment affected immune cell subpopulations in the visceral adipose tissue similarly in WT and *T-bet* ^*−/−*^ mice, with the latter group maintaining their specific reduction in CD3^−^ NKp46+ cells as the development of this cell type is well known to be driven by T-bet expression (Townsend et al., 2004). Whereas microbiota depletion, by the administration of broad spectrum antibiotics, decreased the number of multiple immune cell subpopulations within adipose tissue, in contrast, and consistent with the findings of others in WT mice, the immune cell numbers were increased in the colon (Reikvam et al., 2011). Notably, *T-bet* ^*−/−*^ mice displayed a greater increase in CD3^+^ CD4^+^ and CD3^+^ CD8^+^ cells following antibiotic treatment, compared with WT mice, which may play a role in the greater insulin sensitivity that was observed in this genotype.

These differences in adipose and colonic immune cell subpopulations, both between genotypes and with antibiotic treatment, highlight the importance of crosstalk between the gut microbiota and the immune system and the immune system to the gut microbes. Bi-directionality is thought to exist *in vivo* from the earliest stages of the developing immune system to the influence of the gut microbiota on adipose tissue, colonic and systemic immunity (Maynard et al., 2012). Our observation that global deletion the immune cell transcription factor, T-bet, results in different gut microbiota compared to that of WT mice is strong support that the immune system can regulate the gut microbiota. It is likely that the altered microbiota then impacts the host’s immune system.

We found that inoculation of WT antibiotic-treated mice with gut microbiota from normal (WT) or insulin sensitive (*T-bet* ^*−/−*^) donors is possible by performing fecal gavage and this can impact insulin sensitivity of the WT recipient. However, the effects were temporary, as the composition of the recipient microbiota changed with time and returned to its basal state (Ellekilde et al., 2014). Nevertheless, in this proof-of-concept study we were able to temporarily alter the colonic microbiota, and fecal SCFA composition in a way that had a mild and temporary but significant improvement in the insulin sensitivity of WT mice.

In this study, and our previous work (Stolarczyk et al., 2013), we have found that *T-bet* ^*−/−*^ mice are more insulin sensitive than WT mice. We now show that *T-bet* ^*−/−*^ mice also have altered microbiota with increased fecal butyrate content in addition to the reduced visceral adipose tissue inflammation observed in our previous report (Stolarczyk et al., 2013). It remains to be elucidated which organs are rendered more insulin sensitive in this model. As T-bet expression is restricted to immune cells, it is not possible to do a tissue specific deletion of T-bet to answer this question, as there is a complex network of immune cells across all tissues. Indeed, in metabolically active tissues there is crosstalk between resident immune cells and the parenchymal cells. Differential insulin sensitivity across tissues could be addressed in future work by, for example, undertaking hyperinsulinaemic euglycaemic clamp studies or *ex vivo* studies exploring changes in the classical insulin signalling pathway in insulin sensitive tissues such as adipose, muscle and liver. Furthermore, as T-bet is expressed in a wide number of immune cell subpopulations, specific immune cell deletion of T-bet (e.g., in regulatory T cells) would address which immune cells are key to the metabolic phenotype observed in the global T-bet deletion model.

Although our findings indicate that *T-bet* ^*−/−*^ mice have increased fecal butyrate content, likely from their altered microbiota compared with WT mice, demonstration of a causal link between the increased observed butyrate production and the enhanced insulin sensitivity of *T-bet* ^*−/−*^ mice remains to be determined. Findings from murine studies may not always translate to similar effects in humans, not least because most pre-clinical studies are performed on a single genetic background with few confounding factors impacting experimental design. The *T-bet* gene (*Tbx21*) is highly conserved between mice and humans, as are its target genes (Henderson et al., 2021) and many pathways in obesity and metabolism. However, although many murine studies have shown butyrate to have a role in energy homeostasis, including improving obesity-related glucose metabolism, not all have consistently found a link, particularly in humans (Peng et al., 2023). Indeed, the mechanism of action whereby butyrate influences systemic insulin sensitivity is still unclear. Gut-produced butyrate is primarily absorbed and used by colonocytes, with only a small fraction entering the circulation - the rest is lost in the feces. Circulating levels can, however, be raised by colonic infusion or oral delivery. The role of butyrate in energy homeostasis has recently been comprehensively reviewed (Peng et al., 2023). Butyrate, through its binding of GPCRs, such as FFAR2 and FFAR3, has been shown to affect adipocytes, pancreatic beta cells, enteroendocrine cells, the sympathetic nervous system, colonic tight junctions and immune cell populations including regulatory T cells, as already discussed. The impact of butyrate on glucose homeostasis is likely to involve both local intestinal as well as systemic effects, either directly or indirectly.

In conclusion, we demonstrate the importance of T-bet expression on the regulation of energy homeostasis and gut microbiota composition. We previously described the role of T-bet expression in adipose tissue inflammation (Stolarczyk et al., 2013). This study reveals an additional mechanism of on T-bet regulation of colonic microbiota composition and gut immunity which may play a role in the regulation of insulin sensitivity. Further work, such as 16S sequencing or metagenomic profiling is needed to identify and understand the particular bacteria responsible for these effects, as well as studies to determine its translational relevance to humans. These results open new perspectives on the importance of the immune system in the regulation of energy homeostasis. Targeting the T-bet axis may provide a novel therapeutic approach in the treatment of insulin resistance and type 2 diabetes.

## Data Availability

The original contributions presented in the study are included in the article/supplementary material, further inquiries can be directed to the corresponding author.
