# Supplementary figures and images for: Global deletion of the immune cell transcription factor, T-bet, alters gut microbiota and insulin sensitivity in mice
Source: Front Genet. 2024 Nov 27;15:1502832. doi: 10.3389/fgene.2024.1502832 (PMC11631911; doi:10.3389/fgene.2024.1502832)

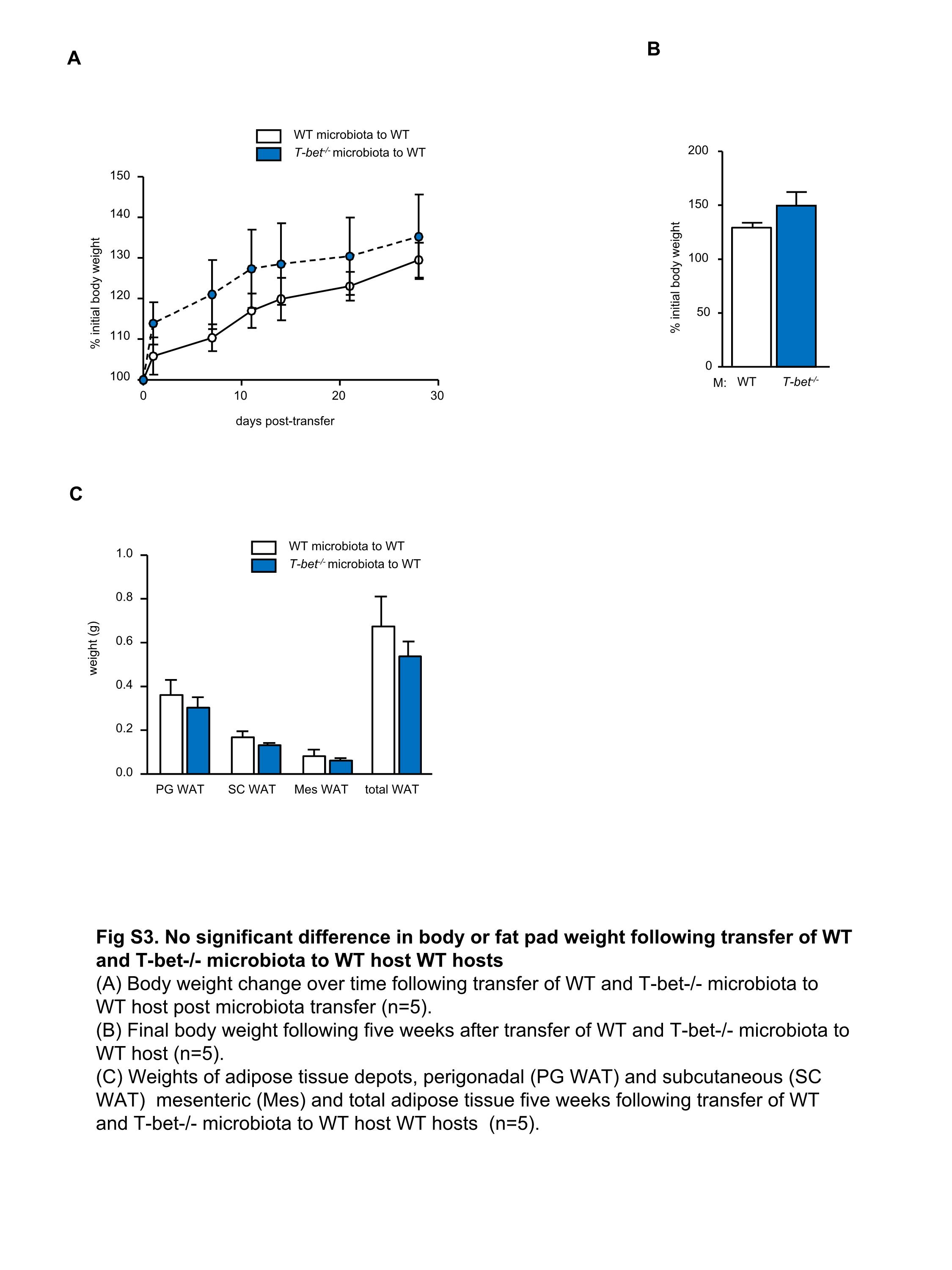

Supplement: Supplementary file 1 [file Image3.jpeg]

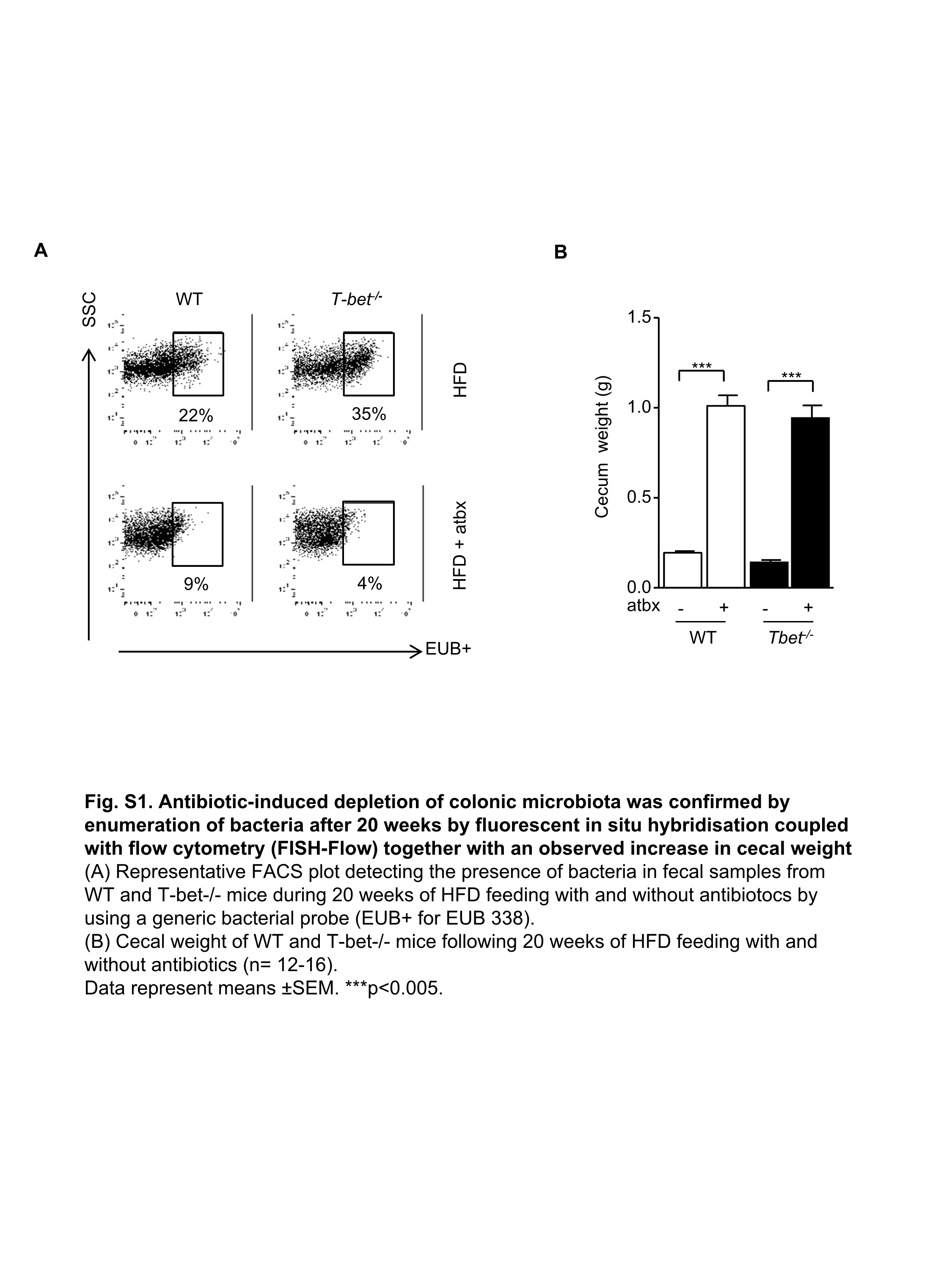

Supplement: Supplementary file 2 [file Image1.jpeg]

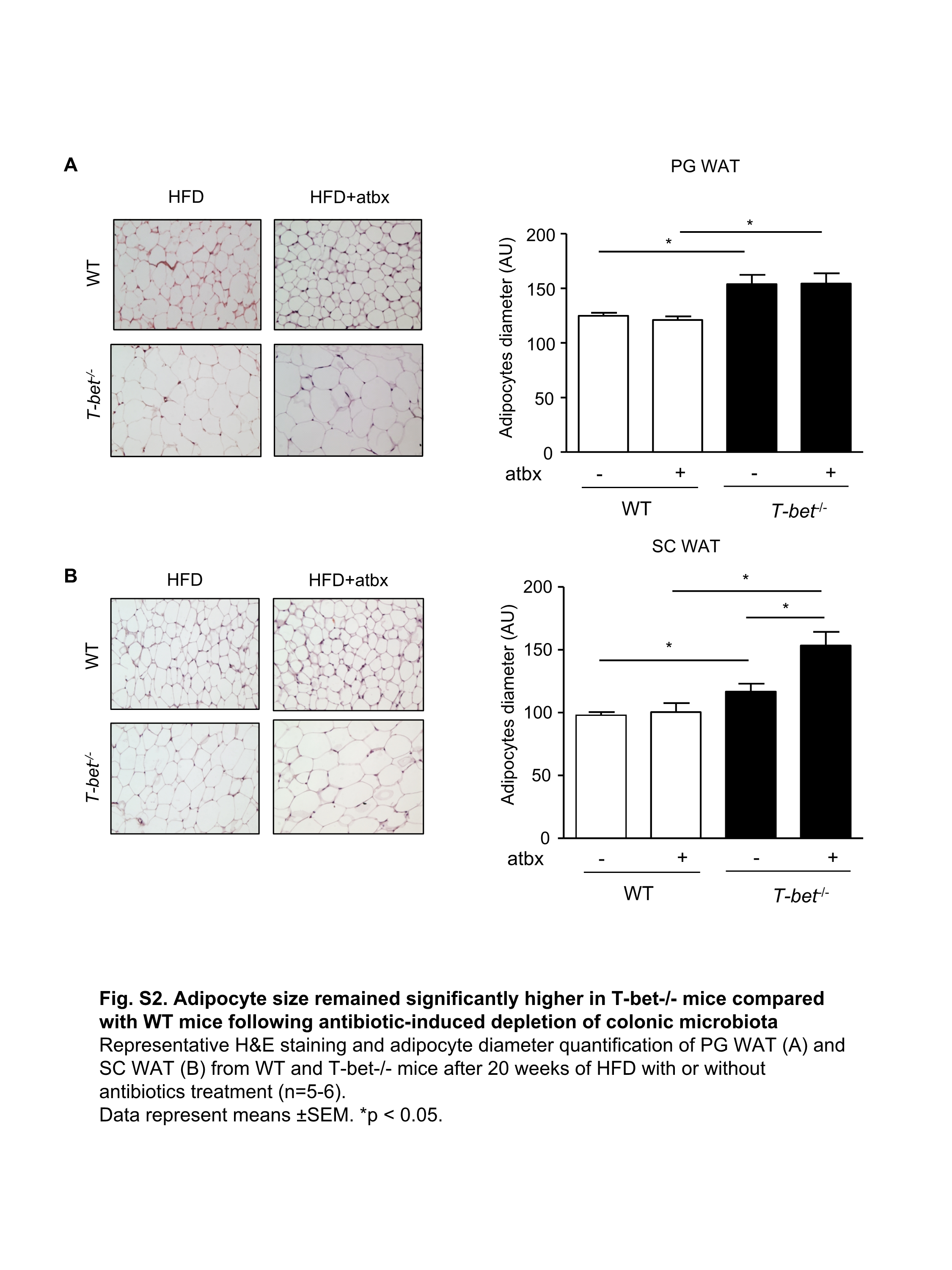

Supplement: Supplementary file 3 [file Image2.jpeg]
